# Induction of DNA breaks and apoptosis in crosslink-hypersensitive V79 cells by the cytostatic drug β-D-glucosyl-ifosfamide mustard

**DOI:** 10.1038/sj.bjc.6600027

**Published:** 2002-01-07

**Authors:** R Becker, A Ritter, U Eichhorn, J Lips, B Bertram, M Wiessler, M Z Zdzienicka, B Kaina

**Affiliations:** Institute of Toxicology, Division of Applied Toxicology, University of Mainz, Obere Zahlbacher Str. 67, D-55131 Mainz, Germany; Division of Molecular Toxicology, German Cancer Research Center, Im Neuenheimer Feld 280, D-69120 Heidelberg, Germany; Leiden University Medical Center, Department of Radiation Genetics and Chemical Mutagenesis, Wassenaarseweg 72, NL-2333 Leiden, The Netherlands

**Keywords:** cyclophosphamide, apoptosis, DNA breaks, cancer therapy, DNA repair

## Abstract

To study molecular aspects of cytotoxicity of the anticancer drug β-D-glucose-ifosfamide mustard we investigated the potential of the agent to induce apoptosis and DNA breakage. Since β-D-glucose-ifosfamide mustard generates DNA interstrand crosslinks, we used as an *in vitro* model system a pair of isogenic Chinese hamster V79 cells differing in their sensitivity to crosslinking agents. CL-V5B cells are dramatically more sensitive (30-fold based on D_10_ values) to the cytotoxic effects of β-D-glucose-ifosfamide mustard as compared to parental V79B cells. After 48 h of pulse-treatment with the agent, sensitive cells but not the resistant parental line undergo apoptosis and necrosis, with apoptosis being the predominant form of cell death (70 and 20% of apoptosis and necrosis, respectively). Apoptosis increased as a function of dose and was accompanied by induction of DNA double-strand breaks in the hypersensitive cells. Furthermore, a strong decline in the level of Bcl-2 protein and activation of caspases-3, -8 and -9 were observed. The resistant parental cells were refractory to all these parameters. Bcl-2 decline in the sensitive cells preceded apoptosis, and transfection-mediated overexpression of Bcl-2 protected at least in part from apoptosis. From the data we hypothesize that non-repaired crosslinks induced by β-D-glucose-ifosfamide mustard are transformed into double-strand breaks which trigger apoptosis via a Bcl-2 dependent pathway.

*British Journal of Cancer* (2002) **86**, 130–135. DOI: 10.1038/sj/bjc/6600027 www.bjcancer.com

© 2002 The Cancer Research Campaign

## 

The newly developed anticancer agent β-D-glucose-ifosfamide mustard (β-D-Glc-IPM) is the glucose-bound form of ifosfamide mustard. The glucose moiety coupled to IPM leads to drug stabilization and preferential uptake of the compound by tumour cells via the Na^+^-D-glucose cotransporter SAAT1 (Veyhl *et al*, 1998). Once inside the cell the drug is cleaved preferentially by intracellular glucosidases to form the ultimate cytostatic drug ifosfamide mustard (Seker *et al*, 1996). It has previously been shown that treatment of MCF-7 cells with β-D-Glc-IPM leads to activation of poly(ADP-ribose) polymerase (Seker *et al*, 2000). In the same study, by using an *in vivo* model system consisting of isogenic cell lines deficient and proficient for either O^6^-methylguanine-DNA methyltransferase (MGMT) or crosslink repair, it could be demonstrated that the most critical cytotoxic lesions caused by β-D-Glc-IPM are very likely to be DNA crosslinks (Seker *et al*, 2000). The cytotoxic pathway evoked by β-D-Glc-IPM has not been elucidated so far. As for many other antineoplastic agents, cytotoxicity of this chemotherapeutic drug could be due to necrosis and/or apoptosis. Apotosis is characterized by a cascade-like activation of intracellular cystein-proteases (i.e. caspases). Caspases pre-exist as zymogens and are activated by proteolytic cleavage by other caspases or by autocatalysis. Distinct caspase cascades are involved in receptor-mediated *vs* chemical-induced apoptosis (Sun *et al*, 1999). Drug-induced apoptosis is often mediated by the mitochondrial pathway leading to activation of the initiator caspase-9 which in turn activates the effector caspases-3 and -7. Active caspase-3 has several substrates, amongst others ICAD (inhibitor of caspase-activated DNase) (Enari *et al*, 1998) and the anti-apoptotic protein Bcl-2 (Cheng *et al*, 1997). Bcl-2 forms heterodimers with the proapoptotic protein Bax and competes with the formation of the channel forming Bax-Bax homodimers. It is hypothesized that the ratio between Bcl-2 and the proapoptotic protein is crucial in determining whether the mitochondria-mediated apoptotic pathway is activated or not (for review, Allen *et al*, 1998). Thus, overexpression of Bcl-2 is expected to result in increased cell survival by preventing cells from undergoing DNA damage-induced apoptosis, whereas decreased expression will favour the onset of the apoptotic machinery. This has indeed been found to be true for several experimental systems in which apoptosis was found to be triggered by non-repaired DNA damage, such as DNA-alkylation repair deficient cells (Ochs and Kaina, 2000).

The aim of the present study was to investigate the mechanistic basis of the cytotoxic effect of the chemotherapeutic drug β-D-Glc-IPM. Since this drug causes cytotoxicity very likely by inducing DNA crosslinks (Seker *et al*, 2000), parameters determining cell death were examined by comparing the response of cells that are hypersensitive to crosslinking agents (CL-V5B cells) with the corresponding parental cell line (V79). We show that CL-5B cells respond with a much higher frequency of apoptosis than the parental cells. Apoptosis was related to DNA breakage, Bcl-2 decline and caspase activation. Therefore, the data support the view that non-repaired DNA crosslinks efficiently trigger the mitochondrial damage pathway via the formation of DNA strand breaks.

## MATERIALS AND METHODS

### Cell culture

CL-V5B cells have been generated by mutagenizing V79 Chinese hamster cells (the line V79B) with ethylnitrosourea and selecting for sensitivity to mitomycin C (Telleman *et al*, 1995). All cells were grown in DMEM/F-12 (Gibco, BRL) containing 5% foetal calf serum. Incubations were at 37°C in a humified 7% CO_2_ atmosphere. Adherent cells were detached with 0.25% trypsin at 37°C.

### Mutagen treatment

Cells were treated for 1 h with β-D-Glc-IPM (Asta-Medica, Frankfurt, Germany) which was freshly solved in PBS by addition of the agent to the medium. After treatment at 37°C, cells were washed with PBS, new medium was added and cells were analyzed at the indicated post-exposure times.

### Colony formation

400–500 cells were seeded into 5-cm dishes. After 6 h, when the cells became adherent, they were treated with β-D-Glc-IPM for 1 h at the indicated concentrations. After incubation, cells were washed with PBS and left for 1 week in the incubator. Colonies were fixed, stained with Giemsa and counted. Each experiment was repeated at least two times.

### Neutral single cell gel electrophoresis (SCGE)

The neutral SCGE (comet assay) was performed as described (Singh *et al*, 1988; Olive *et al*, 1991) with minor modifications. Briefly, following treatment and post-treatment incubation, cells were trypsinized and counted. Ten microlitres of cell suspension (1×10^6^ ml^−1^) were added to 120 μl low melting point agarose (0.5% in PBS, prewarmed to 37°C) and spread onto agarose-coated (1.5% in PBS) slides. Upon cooling for 10 min at 4°C, slides were submerged in pre-cooled lysis buffer (2.5 M NaCl, 100 mM EDTA, 10 mM Tris-HCl pH 7.5, 1% sodium-lauryl sarcosine; shortly before use 1 ml Triton X-100 and 10 ml DMSO per 100 ml were added) for 1 h at 4°C. Cells were electrophoresed at 25 V for exactly 15 min at 4°C in electrophoresis buffer (90 mM Tris-HCl, 90 mM boric acid, 2 mM EDTA, pH 7.5). Slides were washed once in dH_2_O and fixed in ethanol. After drying, cells were stained with ethidium bromide (20 μg ml^−1^) and analyzed microscopically (Olympus BX50, 100× magnification). Olive tail moment was determined by measuring the fluorescence intensity automatically using Kinetic Imaging Komet 4.0.2 software (BFI Optilas, Puchheim, Germany). Extensive control experiments were performed in order to calibrate the experimental conditions for specific detection of DNA double-strand breaks (Lips and Kaina, 2001, and unpublished data).

### Transfection

CL-V5B cells were tranfected with Effectene (Qiagen, Hilden, Germany) according to the manufacturer's protocol. Plasmid pcDNA3.1-myc-his-bcl-2 is a gift from Dr S Dimmeler (Dimmeler *et al*, 1999). Stable transfectants were isolated after 3 weeks of selection in G418 (800 μg ml^−1^) and expanded for further analysis.

### Western blotting

Pelleted cells were washed once in 100 μl PBS and then sonified in PBS on ice in a Branson sonifier. Cell debris were collected by centrifugation at 11 000 r.p.m. and the supernatant further processed. Protein determination was performed using the Bradford reagent (BioRad, Munich, Germany). Fifty micrograms of total protein were loaded onto a 12% SDS gel and, after electrophoresis, transferred to Protran® nitrocellulose membrane (Schleicher and Schüll, Dassel, Germany). After transfer, the membrane was blocked with 5% dry milk in PBS-T, pH 7.6 with 0.2% Tween-20 for 1 h at room temperature (RT). Incubation with the primary antibody and the horse raddish-peroxidase-conjugated secondary antibody was performed in 5% dry milk for 1 h at RT. Washing between each step was performed by shaking the blots three times in PBS-T for 10 min each wash. All antibodies were from Santa Cruz Biotechnology (Heidelberg, Germany). After the final wash, detection was performed by enhanced chemoluminescence (Amersham, Freiburg, Germany). X-ray films were exposed for 1–2 min.

### Detection and quantification of apoptosis

Cells were trypsinized, pooled with cells from the supernatant, centrifuged, washed once with PBS and resuspended in 200 μl binding buffer (100 mM HEPES pH 7.4, 1.4 mM NaCl, 25 mm CaCl_2_, 1% BSA). An aliquot of this suspension was stained with annexin V (Pharmingen, Heidelberg, Germany) and ethidium bromide. Cells were analyzed on a fluorescence activated cell sorter (Becton-Dickinson, Heidelberg, Germany).

### Caspase assay

Caspase assays were performed according to the manufacturer's protocol (R&D Systems, Wiesbaden, Germany) with minor modifications. Briefly, cells were lysed in 100 μl lysis buffer and pipetted in triplicate in 30 μl aliquots onto a 96-well microtiter plate. After addition of reaction buffer and substrate for caspases-3, -8 and -9, respectively, and incubation for 1–3 h at 37°C, extinction was measured in an ELISA reader at 405 nm. Controls were cell lysates containing buffer but without substrate, and substrate containing buffer without cell lysate.

## RESULTS AND DISCUSSION

**Figure 1:**
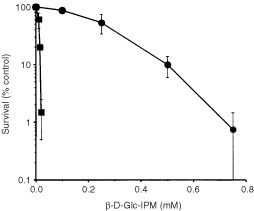
Survival (colony formation) of V79B and CL-V5B cells as a function of dose of β-D-Glc-IPM. Cells were treated for 1 h with the agent and fixed 8 days later. Given are mean values from three experiments ±s.d. •, V79; ▪, CL-V5B.

**Figure 2:**
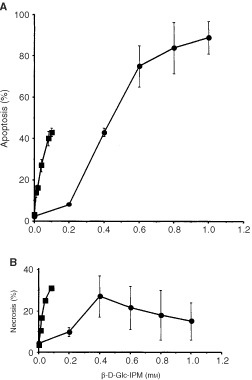
Frequency of apoptosis (**A**) and necrosis (**B**) of V79B and CL-V5B cells as a function of dose of β-D-Glc-IPM. Cells were treated for 1 h and analyzed 72 h post-treatment by FACS. Values are means from three experiments ±s.d. •, V79; ▪, CL-V5B.

**Figure 3:**
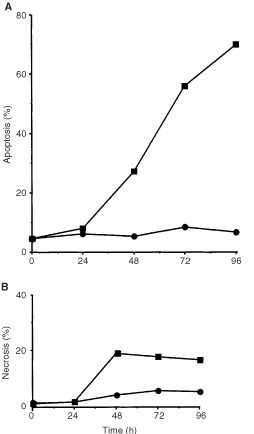
Frequency of apoptosis (**A**) and necrosis (**B**) in V79B and CL-V5B cells as a function of time after pulse-treatment with β-D-Glc-IPM. Exponentially growing cells were treated with a dose of 0.1 mM for 1 h. Apoptosis and necrosis were measured up to 96 h thereafter. Data of one representative experiment are shown. •, V79; ▪, CL-V5B.

The cell line CL-V5B is ∼30-fold more sensitive (based on D_10_ values) than the parental ‘wild-type’ cell line to the cytotoxic effect of β-D-Glc-IPM, as measured by loss of colony-forming ability of the cells (Seker *et al*, 2000 and Figure 1

). To see whether this hypersensitivity is due to the induction of apoptosis and/or necrosis, both endpoints were measured in parallel by annexin V and propidium iodide double-staining and flow cytometry as previously described (Ochs and Kaina, 2000). As shown by the dose-response curves of Figure 2

, the frequencies of apoptosis and necrosis were enhanced in CL-V5B cells compared to the wild-type indicating that both apoptosis and necrosis can be induced in response to β-D-Glc-IPM. This is very likely to be due to the formation of DNA crosslinks to which the cells are hypersensitive (Telleman *et al*, 1995). The time-course of induction revealed that apoptosis occurred not earlier than 2 days after treatment of cells with the drug and further increased during the post-incubation time up to 70% (Figure 3A

). In contrast to this, the frequency of necrosis did not exceed 20% and remained rather constant (Figure 3B). Therefore, apoptosis appears to be the predominant route of cell killing in CL-V5B cells treated with β-D-Glc-IPM.

**Figure 4:**
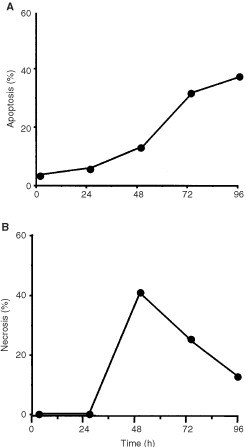
Frequency of apoptosis (**A**) and necrosis (**B**) in wild-type V79B cells as a function of time after treatment with β-D-Glc-IPM with a dose of 0.4 mM for 1 h.

In the time-course experiments shown in Figure 3 the drug concentration was 0.1 mM, which was ineffective to significantly induce apoptosis and necrosis in V79B cells. However, increase of the dose to 0.4 mM also induced apoptosis and necrosis in the parental cells (Figure 4A,B

). This demonstrates that V79B cells basically react in the same way as the hypersensitive cells do, albeit at higher dose level.

**Figure 5:**
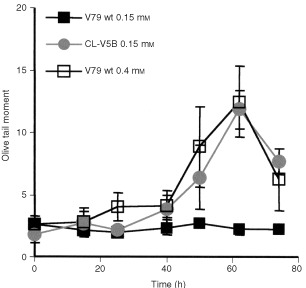
Induction of DNA double-strand breaks in V79B (V79wt) and CL-V5B. Subconfluent cells were pulse-treated for 1 h with 0.15 mM (CL-V5B) or 0.15 and 0.4 mM (V79wt) β-D-Glc-IPM, respectively. Cells were analyzed at the indicated times after treatment by neutral SCGE and olive tail moment is given as mean value ±s.d. For each measure point 100 cells were scored.

Previously we demonstrated that DNA double-strand breaks (DSBs) trigger very effectively apoptosis (Lips and Kaina, 2001). Furthermore, we proposed that, in the case of methylating agent- and UV light-induced apoptosis, DSBs derived from critical primary lesions activate the apoptotic pathway (Ochs and Kaina, 2000; Ochs *et al*, 2001; Dunkern *et al*, 2001a,b). To elucidate whether DSBs are formed in β-D-Glc-IPM treated cells and whether parental V79B and CL-V5B cells respond differently, DSBs were quantified by means of neutral single cell gel electrophoresis (SCGE). As shown in Figure 5

, DSBs are formed in CL-V5B but not in V79B cells upon treatment with a dose of 0.15 mM which is highly effective in inducing apoptosis in CL-V5B but not in the V79B cells. With the equitoxic dose of 0.4 mM (for comparison see Figures 2 and 4) DSBs were also generated in V79B cells which is in accordance with the induction of apoptosis (and necrosis) in these cells under these treatment conditions (Figure 5).

**Figure 6:**
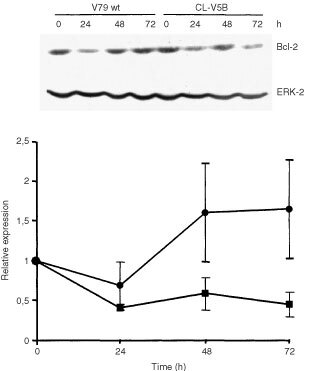
Time course of Bcl-2 expression upon treatment of V79B and CL-V5B cells with 0.1 mM β-D-Glc-IPM. Western blot analysis (a representative example is shown on top) and quantification of Bcl-2 expression (bottom). Bcl-2 expression was normalized for the expression of ERK-2 and shown as x-fold increase over the control level. Quantitative data are from three experiments ±s.d. •, V79; ▪, CL-V5B.

**Figure 7:**
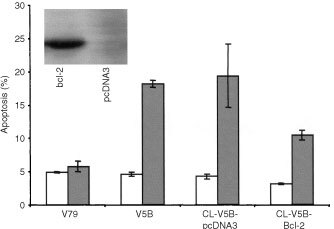
Frequency of apoptosis in IPM-treated (▪) and untreated (□) CL-V5B cells transfected with pcDNA3 vector alone or with myc-his-tagged bcl-2. Insert: Western blot showing the strong expression Bcl-2 and a mock-transfected clone. Given are mean values from four experiments ±s.d.

In various cell systems Bcl-2 has been shown to be critically involved in DNA damage-induced apoptosis. To see whether β-D-Glc-IPM affects Bcl-2 expression, we measured the Bcl-2 level in V79B and CL-V5B cells at various time points after treatment. As shown in Figure 6

, Bcl-2 expression initially decreased in both cell types after treatment. However, after 24 h Bcl-2 in CL-V5B cells remained at a reduced level, whereas the expression recovered in V79B parental cells exceeding even the control level. Since Bcl-2 decline was observed already 24 h after treatment, it clearly preceded the appearance of apoptotic cells indicating that both parameters are interrelated. In order to define more precisely a critical role of Bcl-2 in modulating the apoptotic pathway, we stably transfected CL-V5B cells with a Bcl-2 expression plasmid. As shown in Figure 7

, apoptotic cell death induced by β-D-Glc-IPM was significantly reduced compared to cells transfected with the vector only. This indicates that Bcl-2 decline is causally involved in β-D-Glc-IPM induced apoptosis. The critical role of Bcl-2 in β-D-Glc-IPM-induced apoptosis is in line with data obtained with other repair deficient cell types we have investigated so far. For example, cells deficient for the repair protein O^6^-methylguanine-DNA methyltransferase (MGMT), which are much more sensitive than MGMT proficient cells to O^6^-methylguanine generating agents, show dose-dependently decrease in the Bcl-2 expression level (Ochs and Kaina, 2000). Also, base excision repair defective mouse cells and nucleotide excision repair defective CHO cells responded with Bcl-2 decline upon alkylating agent and UV light as well as cisplatin treatment respectively (Dunkern *et al*, 2001a, b; Ochs *et al*, submitted). Furthermore, electroporation of cells with the restriction endonuclease *Pvu*II inducing DSBs caused Bcl-2 decline that preceded apoptosis (Lips and Kaina, 2001). Taken together the data strongly suggest that Bcl-2 decline is a common feature of DNA damage-triggered apoptosis in fibroblasts.

**Figure 8:**
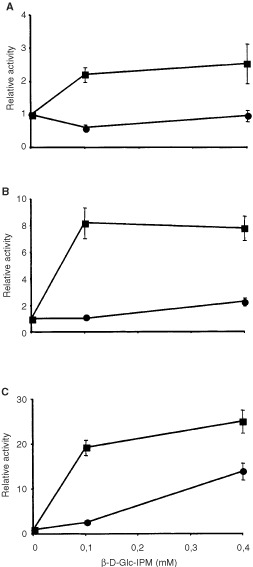
Activation of caspase-3 (**A**), caspase-8 (**B**) and caspase-9 (**C**) as a function of dose. Cells were pulse-treated with β-D-Glc-IPM for 1 h. Capase activity was measured 72 h after treatment. Activities are shown as x-fold increase over control levels. Given are mean values from three experiments ±s.d. •, V79B; ▪, CL-V5B.

**Figure 9:**
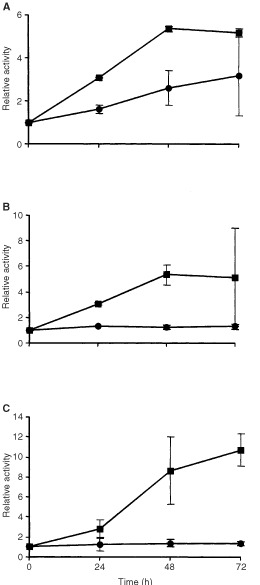
Time course of activation of caspase-3 (**A**), caspase-8 (**B**) and caspase-9 (**C**) upon treatment of V79 and CL-V5B cells with 0.1 mM) β-D-Glc-IPM. Activities are shown as x-fold increase over control levels. Given are mean values from three experiments ±s.d. •, V79B; ▪, CL-V5B.

To further elucidate the molecular mechanism of apoptosis, we investigated the activation of caspases-3, -8 and -9, which are the major executioners of drug-induced apoptosis in many cell systems. As shown in Figure 8

all three caspases were dose-dependently activated in the sensitive CL-V5B cells. Time course experiments revealed that caspase activation occurred 48 h after treatment with a maximum at 72 h (Figure 9

). Strongest induction of activity was found for caspase-9 (10-fold). At the highest concentration of β-D-Glu-IPM applied (0.4 mM) caspase-9 was activated by a factor of 25 and 10 in CL-V5B and V79B cells, respectively. No significant increase in caspase activity was detected in the resistant parental cell line at a concentration of 0.1 mM. Therefore the activation of caspase-9 seems to reflect the induction of apoptosis in these cells (compare with Figures 2 and 4). It should be noted however that unlike DSBs, for which V79B and CL-V5B cells respond similar at equitoxic doses, activation of caspases at the respective doses is much weaker in V79B as compared to CL-V5B cells. Also, caspase activation appears not to be a linear function of dose as compared to the frequency of apoptosis. This could be a reflection of the amplification-based character of activation of caspases and caspase related functions (Bratton *et al*, 2000). The time course and extent of caspase activation suggests a sequential activation, beginning with the upstream initiator caspase-9 followed by caspase-3. Interestingly, caspase-8 was also activated upon drug treatment. While it is well established that caspase-8 is activated via the Fas/CD95 receptor pathway, it has recently been shown that caspase-8 activation can also occur receptor-independently upon treatment with anticancer drugs (Wesselborg *et al*, 1999). Whether or not the CD95 receptor is involved in triggering caspase-8 activation in CL-V5B cells is currently under investigation.

Taken together, our data show that the anticancer drug β-D-Glc-IPM induces cell killing by activating the apoptotic pathway. Similarly to data obtained with cisplatin (Dunkern *et al*, 2001b) it is hypothesized that primary DNA crosslinking lesions do not act as a direct trigger of the apoptotic response, but DSBs arising from them. Necrosis appears to contribute only as a minor fraction to β-D-Glc-IPM-induced cell death. The results also suggest decline of Bcl-2 to be causally involved activating the mitochondrial damage pathway. Since β-D-Glc-IPM basically acts in the same way as cyclophosphamide and related crosslinking agents, the data may be taken at the same time to gain insight into the mode of cell killing provoked by this highly relevant class of anticancer drugs.
